# Cellular Responses of Human Postural Muscle to Dry Immersion

**DOI:** 10.3389/fphys.2019.00187

**Published:** 2019-03-11

**Authors:** Boris S. Shenkman, Inessa B. Kozlovskaya

**Affiliations:** ^1^ Myology Laboratory, State Scientific Center of Russian Federation – Institute of Biomedical Problems, Moscow, Russia; ^2^ Department of Sensory-Motor Physiology and Countermeasures, State Scientific Center of Russian Federation – Institute of Biomedical Problems, Moscow, Russia

**Keywords:** gravitational unloading, postural muscle, support afferentation, muscle atrophy, muscle stiffness, contractile properties, signaling pathways, plantar mechanical stimulation

## Abstract

Support withdrawal has been currently considered as one of the main factors involved in regulation of the human locomotor system. For last decades, several authors, including the authors of the present paper, have revealed afferent mechanisms of support perception and introduced the concept of the support afferentation system. The so-called “dry immersion” model which was developed in Russia allows for suspension of subjects in water providing the simulation of the mechanical support withdrawal. The present review is a summary of data allowing to appreciate the value of the “dry” immersion model for the purposes of studying cellular responses of human postural muscle to gravitational unloading. These studies corroborated our hypothesis that the removal of support afferentation inactivates the slow motor unit pool which leads to selective inactivation, and subsequent atony and atrophy, of muscle fibers expressing the slow isoform of myosin heavy chain (which constitutes the majority of soleus muscle fibers). Fibers that have lost a significant part of cytoskeletal molecules are incapable of effective actomyosin motor mobilization which leads to lower calcium sensitivity and lower range of maximal tension in permeabilized fibers. Support withdrawal also leads to lower efficiency of protective mechanisms (nitric oxide synthase) and decreased activity of AMP-activated protein kinase. Thus, “dry” immersion studies have already contributed considerably to the gravitational physiology of skeletal muscle.

## Introduction

One of the key spaceflight factors having a considerable effect on the regulation of the human locomotor system is support withdrawal. In recent years, several authors, including the authors of the present paper, have revealed afferent mechanisms controlling support perception and introduced the notion of the support afferentation system (Kozlovskaya et al., 2008). Experiments in spaceflight involving space crew members and animals as well as on-ground simulation studies with volunteers and animals show that support withdrawal has a significant effect on motor control mechanisms, in particular, changing the motor unit pools activation pattern by inactivating slow motor units, and leads to reflex tone decrease in postural and mixed postural/locomotor muscles (Kozlovskaya et al., 1988). Similar experiments show profound skeletal muscle atrophy and the associated change in the rate of protein synthesis and degradation, alterations in the signaling mechanisms of protein turnover regulation, alterations in gene expression, including myosin heavy chain isoforms, and so forth (Baldwin et al., 2013). However, all such alterations were discovered in the course of studies conducted either in spaceflight or in on-ground simulations. Under actual microgravity, apart from support withdrawal, other biomechanical factors exist, including axial unloading and the increased ballistic component of movements. In another analog model (unilateral lower limb suspension), human contralateral limbs bear the whole weight, and tendon tension in the unloaded limb is different from tension observed in real microgravity (Lee et al., 2006). In head-down tilt bed rest studies, the support afferentation is not completely withdrawn. The virtually complete removal of support afferentation can only be attained through full submergence of the body in water, i.e., under the conditions of water immersion. The so-called “dry immersion” model which was developed in Russia (Shulzhenko and Vil-Vilyams, 1976; Navasiolava et al., 2011) allows for suspension of subjects in water for a particular period of time (3–56 days) while avoiding negative effects of prolonged water exposure on human skin. Under the conditions of dry immersion postural muscles exhibited decreased maximal voluntary and evoked maximal force and reflectory tone (Kozlovskaya et al., 1988; Grigor’ev et al., 2004), as well as alterations in motor unit recruitment and the parameters of spinal reflexes (Kirenskaia et al., 1986; Sugajima et al., 1996; Shigueva et al., 2015).

Evidence provided by studies on molecular and cellular mechanisms of postural muscle plasticity under gravitational unloading have been thoroughly reviewed in recent years (Kachaeva and Shenkman, 2012; Baldwin et al., 2013; Bodine, 2013; Ohira et al., 2015). However, these studies are focused on the properties of molecular mechanisms regulating the intensity of anabolic and proteolytic pathways in muscle fibers and alterations in contractile responses in muscle fibers of different types as well. It is taken for granted that all effects of microgravity are induced either by disuse of postural muscles or by the removal of resistive component of motor activities, which is often summarized as the “strain vs. activity” problem (Falempin and Mounier, 1998; Ohira et al., 2015). At the same time, these reviews do not cover integrative mechanisms leading to the inactivation of muscle fibers under microgravity. Dry immersion studies involving humans finally give us the possibility to understand the role of support afferentation withdrawal in the development of the three basic components of muscle plasticity under the conditions of real or simulated microgravity, namely hypogravity-induced atrophy (i.e., loss of muscle mass), hypogravity-induced atonia (i.e., sharp decline in stiffness of the whole muscle and single muscle fibers), and alterations in myosin phenotype (alterations in myosin gene expression leading to the domination of fast myosin heavy chain isoform expression).

Also, dry immersion studies allow for detection of the mechanisms that trigger atrophy and atony events at the earliest stages of gravitational unloading.

## Atrophy Development

The first evidence of muscle mass loss induced by real microgravity was obtained in animals. For instance, in rats, 12–14-day flights aboard biosatellites or in space vehicles (the Space Shuttle) led to 35–40% decrease in soleus muscle mass and 15–20% decrease in fast muscles mass (Baldwin et al., 2013). Also, cross-sectional area (CSA) of slow-twitch muscle fibers (in soleus muscle) decreased by over 40% after spaceflight (Baldwin et al., 2013). In rhesus macaques (*Macaca mulatta*), 12–14-day flights led to 30–40% decrease in soleus muscle fiber size and comparable decrease in vastus lateralis muscle fiber size according to our findings (Belozerova et al., 2003; Shenkman et al., 2003). The aim of these experiments, along with the measurement of muscle fiber atrophy in monkeys after spaceflight, was also to compare alterations in the structure and several other properties of muscle fibers in monkeys induced by real microgravity in spaceflight and on-ground model (a flight simulator capsule). Thus, the aforementioned studies provided for the first time the analysis of the effects of various spaceflight factors on atrophic changes in postural soleus muscle and nonpostural vastus lateralis muscle fibers. It was found that the profound atrophy of soleus muscle fibers occurred only in animals exposed to the conditions of real spaceflight, while atrophic changes in vastus lateralis muscle occurred in animals under the conditions of both real spaceflight and on-ground flight simulator capsule. Thus, the evidence showed that while the atrophy of soleus muscle was mainly induced by the removal of terrestrial gravity, the atrophy of vastus lateralis muscle was, at least partially, induced by its decreased contractile activity due to restricted mobility in the space vehicle.

The study of astronauts’ skeletal muscle tissue before and after spaceflight was initially impeded by the fact that under real spaceflight conditions, astronauts are subjected not only to microgravity but also to physical exercises utilized as a countermeasure to adverse spaceflight effects. Moreover, the protocols of such exercises could not be standardized for a long time. The research team led by V. R. Edgerton was the first to analyze the vastus lateralis muscle tissue of three astronauts after a 5-day flight and of five astronauts after an 11-day flight aboard Space Shuttle crafts (Edgerton et al., 1995). After 5 days of flight, the range of alterations in the muscle fiber CSA was 11% for slow-twitch muscles and 24% for fast-twitch muscles. After 11 days of flight, the alteration range was 16–36%, with the fast-twitch muscle atrophy also more profound in this case.

We were able to analyze for the first time human vastus lateralis muscle fiber after 3-day and 7-day “dry” immersion (Shenkman et al., 1999). After 3 days of immersion, the evidence reliably showed a 7.5% decrease in CSA of slow-twitch muscle fibers, with the 8.9% (*p* < 0.05) decrease for the fast-twitch muscles. After 7 days of immersion, the decrease of the slow-twitch muscle fiber CSA was by 17.3%, with 15.3% for the fast-twitch muscles. Comparison of this evidence with that obtained by the Edgerton team shows that the CSA alteration ranges under immersion and in flight over a short period of time differ insignificantly and that the atrophic alterations in the slow and fast-twitch muscle fiber CSA (with even a slightly more profound changes in fast-twitch muscle, especially in flight) do not significantly differ both under real microgravity and immersion.

Later, we were able to obtain evidence of the decrease in human soleus muscle fiber CSA (Grigor’ev et al., 2004; Shenkman et al., 2004). After 7-day immersion, without exposure to other factors, we observed a decrease in the slow-twitch muscle fiber CSA by, on average, 24%, while no significant alterations in the fast-twitch muscle fiber CSA occurred. These alterations are difficult to compare with the similar data obtained by Widrick et al. (1999) and Trappe et al. (2009), who studied the Shuttle Vehicle and International Space Station crew members, due to the obviously different durations and conditions of exposure. Additionally, some of the latter studies involved astronauts performing 90-day and even longer flights with a significant amount of in-flight physical exercise.

It is desirable to compare the data that we obtained in immersion studies with the data on muscle fiber size recorded in head-down tilt (HDT) experiments of various durations. The atrophic alteration values found in our studies after 7 days of immersion were usually recorded on the 14th, 30th, or 60th day of the HDT (Hikida et al., 1989; Bamman et al., 1998; Clarke et al., 1998; Shenkman et al., 2000). Sadly, we could not find publications containing measurements of muscle fibers recorded during shorter-term HDT bed rest studies. Comparison of muscle fiber measurements made in immersion studies with data obtained in unilateral lower limb suspension (ULLS) studies shows the following. Alterations in the morphological characteristics of soleus and vastus lateralis muscle fibers after short-term exposure to ULLS are less profound compared to those after 7-day dry immersion (Hather et al., 1992; Brocca et al., 2015). After 14-day ULLS, no alterations in vastus lateralis muscle fiber size were found (Deschenes et al., 2002). Furthermore, the distribution of alterations in various muscles differs from such found under hypokinesia or immersion. In ULLS, a decrease in vastus lateralis muscle fibers CAS is more pronounced than that of soleus muscle (Hackney and Ploutz-Snyder, 2012). It can be suggested that in ULLS model, the functional unloading for soleus muscle is less pronounced than that for other muscles. This suggestion is confirmed by studies of functional activity of the portions of the Achilles tendon using functional MRI. Evidently, the strain in the middle portion of the Achilles tendon does not decrease under exposure to ULLS (Lee et al., 2006). Consequently, we can conclude that, for unknown reasons, the extent of soleus muscle unloading in ULLS is less pronounced than in spaceflight.

Thus, after 3–7 days of “dry” immersion rapid atrophic alterations occur that are similar to those found in astronauts after short-term spaceflights (at least in vastus lateralis muscle). This phenomenon can be described as rapid atrophy and suggesting that the removal of support afferentation triggers rapid and profound changes in postural and mixed postural/locomotor muscles. Also worthy of note is the fact that, as opposed to the rarely described effects of short-term bed rest and unilateral lower limb suspension, soleus muscle atrophy after 7-day immersion is only significant in slow-twitch muscle fibers, which suggests a direct dependence of the state of these fibers on the intensity of afferent signaling.

## Contractile Properties of Single Permeabilized Fibers

Animal experiments (rats and monkeys) have repeatedly shown that exposure to real microgravity for 7–14 days leads to decreased contractile properties of single permeabilized fibers (Holy and Mounier, 1991; Stevens et al., 1993; Fitts et al., 2000). These experiments demonstrated a decrease in soleus muscle fibers maximal tension (sometimes decrease in specific tension), the decrease of calcium sensitivity (the right-hand shift of the Ca-tension curve), and the increase of the unloaded shortening velocity. Studies of muscle fiber physiology in astronauts began in the late 20th century. The shortest spaceflight analyzed by researchers lasted 17 days. Analysis of the contractile properties of permeabilized and calcium-stimulated fibers showed that maximal tension decreased by (on average) 20%, while the unloaded shortening velocity increased by 30% (Widrick et al., 1999). It is worth noting that, unlike experiments on animals exposed to real or simulated microgravity, the aforementioned study did not reveal alterations in calcium sensitivity in humans. The pronounced shift of the Ca-tension curve was only recorded for one crew member whose muscle fibers exhibited the largest decrease in maximal tension. According to the same researchers, a decrease in maximal tension in human soleus muscle fibers following 17-day bed rest did not exceed 13% (Widrick et al., 1997).

**Figure 1:**
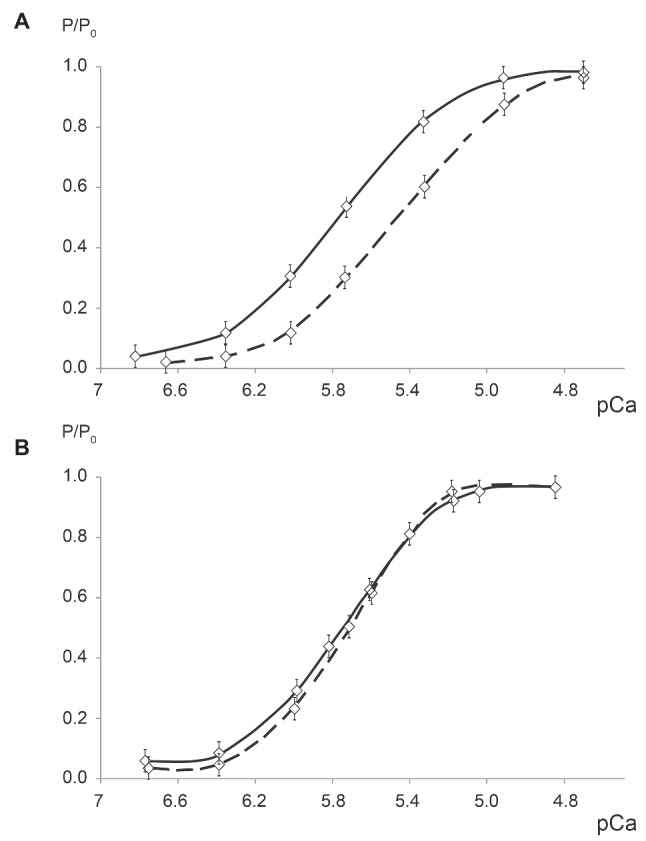
Calcium-tension relationships in permeabilized fibers of human soleus before and after 7-day dry immersion with **(A)** and without **(B)** plantar mechanical stimulation (Shenkman et al., 2004). With kind permission from NAUKA Publishing House (Official Publishers of BIOFIZIKA journal).

In 2002 and 2009, we studied the effect of 7-day “dry” immersion on the contractile properties of human soleus muscle fibers. In the first series of experiments, maximal tension of soleus muscle fibers decreased by 32% (Shenkman et al., 2004), and in the second series of experiments, it decreased by 26% (Ogneva et al., 2011a,b). No significant alterations in specific tension were noted. Both series of experiments exhibited consistent and significant right-hand shifts of the Ca-tension curve (according to рСа50) which indicates the sufficient decrease in calcium sensitivity of the myofibrillar apparatus (Figure 1). Note that in both series of experiments, alterations in the aforementioned parameters were more pronounced than those exhibited by astronauts although the duration of exposure was approximately 2.5 times shorter in the former case. The similar result had been reported in relation to isokinetic dynamometry studies conducted before and after a 7-day spaceflight and a 7-day “dry” immersion (Kozlovskaia et al., 1984). The decrease in maximal voluntary strength was found to be more pronounced under the conditions of immersion than in spaceflight. The authors explained these unexpected results by the fact that while the amount of contractile activity in human lower limb muscles is smaller in spaceflight than on the ground (although the authors of the latter paper do not provide exact data on the amount of contractile muscle activity in spaceflight), it is nevertheless still larger in spaceflight than under the conditions of immersion hypokinesia. This suggestion has recently been corroborated by the data obtained from the study of the crew members’ physical activity in spaceflight (Fraser et al., 2012). The larger decrease in the contractile properties of human single soleus muscle fibers under the conditions of immersion than in spaceflight may be accounted for by the similar suggestion.

## Transversal Stiffness of Myofibrillar Apparatus

We used atomic force microscopy for the first study of the transversal stiffness of the various compartments of human soleus muscle fibers after 7 days of “dry” immersion (Ogneva et al., 2011a,b). The measurements were performed according to the original protocol (Ogneva et al., 2010) on single permeabilized fibers in the relaxed state and at the peak of isometric activity with *pCa* = 4.2. Some of the fibers were treated with Triton X-100 to lyse membrane structures and make the myofibrillar apparatus accessible to the cantilever. After the immersion, in relaxed fibers, the myofibrillar stiffness decreased twofold in the region of the Z-disc and threefold in the region of the M-disc. Between the Z-disc and the M-disc (the so-called half-sarcomere region), myofibrillar stiffness decreased more than twofold. Our experiment showed the approximately identical decrease of active fiber stiffness in all the regions of the myofibrillar apparatus that was examined: up to 0.4-fold of its initial value. Thus, the differences between the stiffness values in relaxed and active fibers were less significant after the immersion than before it. This evidence is in accordance with the measurements of the contractile properties of permeabilized fibers (see above) which show the decrease of both maximal isometric tension and calcium sensitivity in muscle fibers. It may be suggested that exposure to “dry” immersion leads to the malformation of cross bridges in calcium-activated fibers. To understand the causes of this phenomenon, it is necessary to analyze the effects of support withdrawal on myofibrillar cytoskeletal proteins.

## Myofibrillar Cytoskeletal Proteins

**Figure 2:**
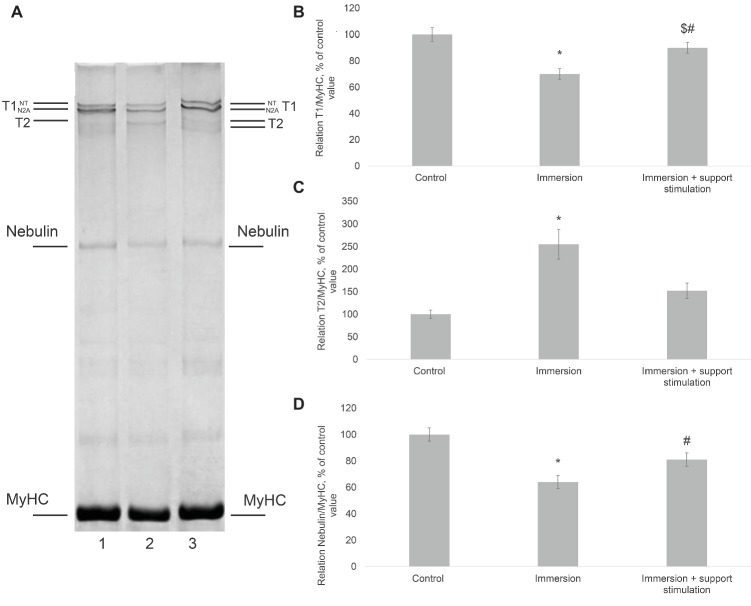
Changes of titin and nebulin content in human soleus muscle. **(A)** SDS-PAGE analysis of titin and nebulin content in human soleus muscle. 1. Control, 2. Immersion, 3. Immersion + support stimulation. MyHC—myosin heavy chains, T2 is proteolytic fragment of intact titin-1 (T1). N2A and NT are isoforms of T1. **(B)**, **(C)**, and **(D)** Densitometric quantification plots of the titin T1, titin T2, and nebulin, respectively (Shenkman et al., 2004). With kind permission from NAUKA Publishing House (Official Publishers of BIOFIZIKA journal).

Titin is the chief scaffold protein connecting the basic contractile and regulatory elements of the sarcomere (thin and thick filaments, Z-discs and M-discs) and, according to recent research, acting as mechanosensor in muscle fibers (Lange et al., 2005). Decrease in titin content under unloading was first discovered in rats by Kasper and Xun (2000). Similar evidence was found at the Lille University (Toursel et al., 2002). Also in that year, we found decrease in titin content and increase in the content of its proteolytic fragment, T2, in rat soleus muscle after 14-day unloading (Shenkman et al., 2002). Since titin is considered to be a part of the series elastic component and decline in fiber stiffness is observed as early as after 3 days of hindlimb unloading (Ogneva, 2010), its breakdown or increase in elasticity could be expected already after 2 or 3 days of unloading. Apparently, however, this is not the case. Studies conducted in collaboration between our laboratory and Podlubnaya group in 2008 (Ponomareva et al., 2008) showed that the basic titin isoform, N2A, typical for skeletal muscles, remains intact after 3 days of unloading. Earlier, finding no changes in titin content after 3 days of unloading, Goto had discovered decrease, rather than increase, in elasticity in the elastic region of the titin molecule located between the Z-disc and the N2A domain (including the spring PEVK region) after such unloading (Goto et al., 2003). Recently, the explanation of this phenomenon has been suggested as a result of several studies (Nishikawa et al., 2012), which showed that increase in calcium ions content in fibers [occurring under gravitational unloading (Ingalls et al., 1999, 2001; Shenkman and Nemirovskaya, 2008)] leads to the strong binding of titin N2A domain to thin filaments. Even more recently, a 40% decrease in titin content and an increase in the T2 proteolytic fragment content were found to have occurred in mice gastrocnemius medialis muscle after a 30-day spaceflight on the Bion-M1 satellite (Ulanova et al., 2015). The same study provided the first evidence of a significant increase in titin phosphorylation level. Sharp decrease in titin content and increase in T2 content was first discovered after 7-day “dry” immersion in human soleus muscle (Figure 2; Shenkman et al., 2004). Experiments on single permeabilized fiber specimens show that partial titin breakdown is caused by the activity of calpains (calcium-dependent cysteine proteases), particularly μ-calpain (Murphy et al., 2006). Studies on passive single muscle stiffness in animals show that titin has a crucial role in determining the elastic properties of muscular tissue (Fukuda et al., 2008). Consequently, it may be suggested that decrease in the intrinsic muscle stiffness after “dry” immersion may be partially caused by decreased titin content.

Another effect of “dry” immersion on human soleus muscle is a decrease in the content of nebulin, an important sarcomere skeleton protein acting as scaffold for thin filaments (Figure 2; Shenkman et al., 2004). The function of nebulin is clearly demonstrated by nebulin knockout experiments in which muscle ultrastructure exhibits severe malformation and shorter length of actin filaments and even significant partial loss of thin filaments (Li et al., 2015). Similar malformations of thin filaments were registered by Riley and Fitts in their studies of human soleus muscle biopsy samples after 17-day spaceflight and 17-day bed rest (Riley et al., 1998). The authors note that alterations in thin filaments are accompanied, under such conditions, by a decrease in maximal tension and increase in unloaded shortening velocity in single permeabilized fibers. It is possible that structural alterations in thin filaments and the corresponding alterations in stiffness of myofibrillar apparatus are caused precisely by significant partial loss of nebulin molecules pool which we discovered in human soleus muscle after “dry” immersion and in gastrocnemius medialis muscle in mice after long-term spaceflight (Ulanova et al., 2015).

After 7-day “dry” immersion soleus muscle samples exhibited a 20% decrease in the content of desmin (Ogneva et al., 2011a,b), a protein interlinking myofibrils in the Z-disc region and other cellular compartments (mitochondria, myonuclei, etc.) (see review by Capetanaki et al., 2007). Earlier decrease in desmin level was shown in unweighting experiments on rats (Enns et al., 2007; Ogneva et al., 2010).

Thus, under the conditions of “dry” immersion, a phenomenon is observed that was discovered in unweighting experiments on rodents: decrease in cytoskeletal protein content in the sarcomere. It primarily concerns elastic giant proteins such as titin and nebulin. Significant partial loss of these proteins, which contribute to passive resistance in strained muscular tissue or in contracted muscle, necessarily affects muscle fiber stiffness (see above). Also, a number of cytoskeletal proteins is involved in the regulation of the actin-myosin interfilament spacing and, consequently, in the formation of cross-bridges. For this reason, contraction in normal muscle fibers, with intact sarcomeric cytoskeleton, cannot be described as fully unloaded even in experiments involving withdrawal of resistive load. Although at present, it is difficult to measure intrinsic resistance in muscles caused by cytoskeletal proteins, we suppose it could have a certain effect on both the contractile properties and signaling regulatory mechanisms. Partial loss of intrinsic resistance observed in gravitational unloading (particularly in “dry” immersion) necessarily affects signaling processes.

## Subsarcolemmal Cytoskeleton and Sarcolemmal Permeability

It has been suggested that a number of mechanosensory events in any cell, including muscle fibers, is localized in the cortical compartment adjacent to the cell membrane (Durieux et al., 2007). Consequently, investigation of the state of cell cortex proteins and the state of the sarcolemma is necessary. Using an atomic-force microscope, we were able to register alterations in sarcolemmal (cortical) cytoskeleton stiffness in the Z-disc and M-disc regions and in the so-called half-sarcomere region (i.e., between the discs) in human soleus muscle after 7-day “dry” immersion. 61.7% stiffness decrease was exhibited in cortical cytoskeleton. While before the immersion calcium activation led to (approximately) 200% increase in cortical cytoskeleton stiffness (apparently, through force transduction from myofibrils to cortical cytoskeleton *via* cytoskeletal network), after 7-day immersion there was only 80–120% increase in active fiber stiffness (Ogneva et al., 2010). However, such a dramatic decrease in cortical cytoskeleton stiffness was not caused by changes in dystrophin content because after immersion no sarcolemmal dystrophin disruptions were significantly exhibited (Gasnikova et al., 2004). Note that a decrease in dystrophin content and the increased number of fibers exhibiting sarcolemmal dystrophin disruptions were observed in rat soleus muscle after 2-week, or longer-term, hindlimb suspension (Chopard et al., 2001; Gasnikova and Shenkman, 2005). It is possible that sarcolemmal dystrophin degradation occurs only under prolonged gravitational unloading. Current studies suggest that dystrophin, having certain stiffness (Gumerson and Michele, 2011), prevents macromolecules from passing through the membrane. However, muscle creatine phosphokinase is found in human blood samples taken from people regularly engaged in moderate physical activity. 7-day exposure to “dry” immersion is significantly known to lower creatine phosphokinase content in blood (Gasnikova et al., 2004). This evidence is in accordance with the evidence provided by a study of creatine phosphokinase content in the blood of astronauts after long-term spaceflights. That study also registered considerable decrease in the content of that enzyme in systemic blood during spaceflight (Markin et al., 1998). Decrease in circulating creatine phosphokinase levels during spaceflight or “dry” immersion may indicate a lesser disruption of sarcolemma and its cytoskeleton, possibly as a result of decrease in muscle fiber contractile activity. Decrease in this muscle contractile activity is also evidenced by electromyographic data (Miller et al., 2004). In another simulated rat hindlimb suspension study, Christine Kasper (Kasper, 1995) showed for the first time that exposure of animals to such conditions leads to lower macromolecular permeability of the sarcolemma; however, during the acute period of recovery after unloading, albumins dyed with Evans blue were seen to intensively penetrate soleus muscle fibers sarcolemma. In our experiments, during the recovery period, an enhanced albumin transport across the sarcolemma was accompanied by increased number of sarcolemmal dystrophin disruptions (Gasnikova and Shenkman, 2005).

Thus, apparently, support withdrawal and postural muscle inactivation under the conditions of immersion can prevent sarcolemmal disruption and lower the intensity of macromolecular transport across the sarcolemma.

## Signaling and Protective Mechanisms

Signaling mechanisms controlling changes in muscle mass and muscle contractility have become the object of intense studies in recent years (for review see Schiaffino et al., 2013). For instance, these studies have elucidated changes in certain signaling pathways in postural soleus muscle. It has been shown, in particular, that subcritical depolarization of the sarcolemma occurs at the early stages of the muscle contractile activity decrease and is caused by decreased electrogenic activity of the α2 isoform of the Na, K-ATPase (Kravtsova et al., 2015). With membrane potential decreased to −40 mV, part of voltage-gated L-type calcium channels may become activated and calcium ions are accumulated in the myoplasm (Ingalls et al., 1999, 2001). Simultaneously, nitric oxide production, heat shock protein content, and calpastatin expression are decreased during unloading (Enns et al., 2007; Lomonosova et al., 2011, 2012). Deficiency of these three signaling factors being inhibitors of calpain activities promotes the μ-calpain activation and cytoskeletal proteins degradation. Additionally, decreased level of nitric oxide leads to ubiquitin ligases gene expression activation (Lomonosova et al., 2011). The E3 ubiquitin ligases expression also intensifies in response to IRS-1 (insulin receptor substrate-1) ubiquitinylation and degradation with subsequent Akt dephosphorylation (Nakao et al., 2009). The Akt dephosphorylation leads to dephosphorylation of FOXO1 and FOXO3 and their translocation into myonuclei. These transcription factors bind DNA to activate the E3 ubiquitin ligases expression. Also early stages of gravitational unloading result in a significant decrease in the rate of protein synthesis, 28S ribosomal RNA expression, dephosphorylation of glycogen synthase kinase 3β (GSK3β) (Mirzoev et al., 2016) as well as increased eukaryotic elongation factor 2 (eEF2) phosphorylation (Krasniy et al., 2013). Recent studies have also showed a decrease in PGC1α gene expression which contributes to activation of ubiquitin ligases gene expression at the early stages of unloading (Cannavino et al., 2014).

As shown above, much has been understood about early events in skeletal muscle in laboratory rodents exposed to gravitational unloading (using hindlimb suspension model). However, there is still a lack of data on signaling mechanisms initiating muscle atrophy in humans during the early stages of unloading. The first data on such events were obtained employing unilateral lower limb suspension which, judging by data on the distribution of atrophic changes among muscles (Hackney and Ploutz-Snyder, 2012), does not completely simulate gravitational unloading under real microgravity. However, short-term limb suspension causes increased ubiquitin ligases gene expression and decreased FAK phosphorylation level (Gustafsson et al., 2010; Flück et al., 2014). These data correspond well with the data obtained earlier in rat studies.

So far, there exist few studies on signaling mechanisms in human soleus muscle after dry immersion. After 7-day dry immersion, a dramatic decrease in neuronal nitric oxide synthase (nNOS) level was observed (Moukhina et al., 2004). More recently, a decrease in the level of this enzyme, although less pronounced, was observed after 3 days of immersion (Vilchinskaya et al., 2015). After 3 days of exposure, decrease in phosphorylation level of this enzyme was also observed (Vilchinskaya et al., 2015). Reduction of the level and degree of nNOS had been observed earlier in suspension studies involving rats and in bed rest studies involving humans (for review see Shenkman et al., 2015). In a 14-day rat suspension experiment we demonstrated a decrease in nitric oxide level in soleus muscle (Lomonosova et al., 2011). More recently we found a strong evidence of nitric oxide level decrease in rat soleus muscle already after 24-h suspension (Vilchinskaya et al., 2015, unpublished observation). It is possible that decrease in nitric oxide level is caused, at least in part, by decreased gene expression, or intensified degradation, of neuronal NO synthase. Nitric oxide is the endogenous inhibitor of calpain activity (Michetti et al., 1995). Consequently, decrease in nitric oxide level may lead to calpain activation (like in rat tail suspension studies—see above) and, accordingly, degradation of certain cytoskeletal proteins. Indeed, after 3 days of immersion, we found a significant decrease in desmin level in human soleus muscle (Vilchinskaya et al., 2015). Among kinases catalyzing NO synthase phosphorylation, we focused on AMP-activated protein kinase (AMPK) (Chen et al., 2000) whose activity is known to increase in response to AMP accumulation (for review see Mounier et al., 2015 which is usually caused by high energy expenditure) and decrease in response to high-energy phosphates phosphorylation [which can be caused by muscle inactivity (Ohira et al., 2015)]. We supposed that under the conditions of short-term “dry” immersion the degree of AMPK phosphorylation may decrease. Indeed, after 3 days of immersion phosphorylated AMPK level decreased significantly (Vilchinskaya et al., 2015). Thus, dry immersion studies provided us with the first evidence of AMPK phosphorylation decrease after short-term unloading. These data were confirmed in rat hindlimb suspension study (Mirzoev et al., 2016). Considering the importance of AMPK in the regulation of energy metabolism, gene expression and protein turnover, it may be suggested that this phenomenon may contribute to triggering a chain of events leading to the formation of the atrophic signaling pattern.

Neuronal NO synthase phosphorylation may also occur in response to the kinases of the IGF-1/Akt signaling pathway (Hinchee-Rodriguez et al., 2013). During the first week of hindlimb suspension rodents exhibited the ubiquitination and degradation of IRS-1 (insulin receptor substrate), which regulates the phosphorylation degree of the downstream protein kinases of the PI3K signaling pathway (Nakao et al., 2009). In our study, after 3 days of “dry” immersion no significant changes in IRS-1 were found (Vilchinskaya et al., 2015). We expect that future studies will show whether AMPK dephosphorylation at the early stages of unloading causes a decrease in nNOS activity and, consequently, a decrease in NO production and calpain activation. These and other aspects of atrophic and atonic signaling under short-term “dry” immersion are still need to be studied.

## The Role of Support Afferentation in the Development of Hypogravity Muscle Syndrome

The direct influence of support afferentation on human locomotor functions was first shown in the Soviet-Cuban joint experiment aboard the Soviet space vehicle. That experiment involved plantar mechanical stimulation (Hernandez-Korwo et al., 1983). In subsequent “dry immersion” studies, a modified plantar stimulation device was used that allowing for a long-term series of stimulation. These studies have shown, in particular, that plantar stimulation under the conditions of immersion allows for maintaining the normal level of electrical activity and reflectory transversal stiffness in soleus muscle (for review see Grigor’ev et al., 2004).

The following protocol has been used during our experiments: plantar stimulation, with pressure equaling 40 kP, was performed every day over the course of 6 h, for 20 min at the beginning of each hour, reproducing two natural modes of locomotion: slow walking (75 steps per min) and fast walking (120 steps per min), each for the duration of 10 min. After 7-day immersion with plantar mechanical stimulation no decrease in slow-twitch muscle fiber cross-sectional area and no discernible shifts in the proportion of fibers expressing slow and fast myosin heavy chain isoforms were observed in soleus muscle (Shenkman et al., 2004). Thus, atrophy development was prevented without usage of intense running or resistance exercise. Plantar stimulation allowed to prevent decrease in maximal isometric tension and calcium sensitivity in permeabilized muscle fibers (Figure 1; Litvinova et al., 2004; Shenkman et al., 2004; Ogneva et al., 2011a,b). These findings give evidence that muscle activity induced by support afferent stimulation could prevent malformation of cross-bridges.

In regard to studies on transversal stiffness of myofibrillar apparatus (using atomic force microscopy with permeabilized fibers pre-treated with Triton X-100), in relaxed fibers after support stimulation under the conditions of 7-day immersion decrease in stiffness was registered only in the Z-disc region (30%). In all the other sarcomere regions no significant alterations in transversal stiffness were registered as compared with pre-immersion values (Ogneva et al., 2011a,b). While the use of support stimulation did not completely prevent stiffness decrease in active fibers (with pCa = 4.2), its range across sarcomere regions was 15–25%. Thus, a decrease in active fiber stiffness was significantly less pronounced after immersion combined with support stimulation than after “pure” immersion (Ogneva et al., 2011a,b). Apparently, muscle activity allowed preserving the stiffness of myofibrillar apparatus by preventing malformations in cross-bridges and degradation of sarcomere cytoskeleton proteins. The latter suggestion is corroborated by data on titin and nebulin content in human soleus muscle after “dry” immersion involving support stimulation. In such immersion studies, members of the group exposed to support stimulation exhibited only a slight decrease in titin and nebulin content, while members of the group that was not exposed to support stimulation exhibited up to 40% decrease in titin and nebulin content (Figure 2; Litvinova et al., 2004; Shenkman et al., 2004). Decrease in desmin content was also not found in subjects exposed to support stimulation. As the degradation of the aforementioned cytoskeletal proteins is usually believed to be caused by μ-calpain, it is possible that muscle activity induced by afferent stimulation initiates the endogenous mechanism of calpain inhibition. Such a mechanism may be involved in maintaining the high level of nitric oxide synthase activity and NO being the endogenous inhibitor of calpain activity (see above). In our study, plantar mechanical stimulation not only prevented a decrease in nNOS content but also contributed to its increase as compared with the pre-immersion level (Moukhina et al., 2004). Further research will show the accuracy or inaccuracy of our hypotheses concerning the mechanism that allows support afferentation to ensure the consistent, albeit low, level of postural soleus muscle activity and to maintain the normal state of cytoskeleton and the systems of actomyosin motor mobilization.

We have also shown that some parameters that had been altered in response to gravitational unloading do not change in response to support stimulation. Thus, in an active muscle fiber, dramatic decrease in sarcolemma stiffness can be completely prevented by support stimulation, apparently, because this parameter depends on force transduction from myofibrils to cortical cytoskeleton. However, the transversal stiffness of relaxed fiber sarcolemma under the conditions of immersion remained decreased even when support stimulation had been performed during immersion (Ogneva et al., 2011a,b). Similarly, despite support stimulation, the content of α-actinin-1, a sarcolemmal cytoskeleton protein, remains decreased (Ogneva et al., 2011a,b). It is possible that the force that muscle fiber generates when exposed to afferent stimulation is insufficient for macromolecular permeability since creatine phosphokinase content in blood also remained decreased under such conditions (Gasnikova et al., 2004).

## Conclusion

Apparently, studies concerning cellular responses of human skeletal muscle to real microgravity (in spaceflight) remain scarce. This makes on-ground studies, especially those involving humans, particularly important. The present review is a summary of data that allows us to appreciate the value of the “dry” immersion model for the purposes of studying cellular responses of human skeletal muscle, particularly postural muscle, to gravitational unloading. The model proves to be especially useful to study the interaction between systemic mechanisms (primarily, ones of the central nervous system) and local (electrophysiological and biomechanical) mechanisms of hypogravity muscle syndrome development.

**Figure 3:**
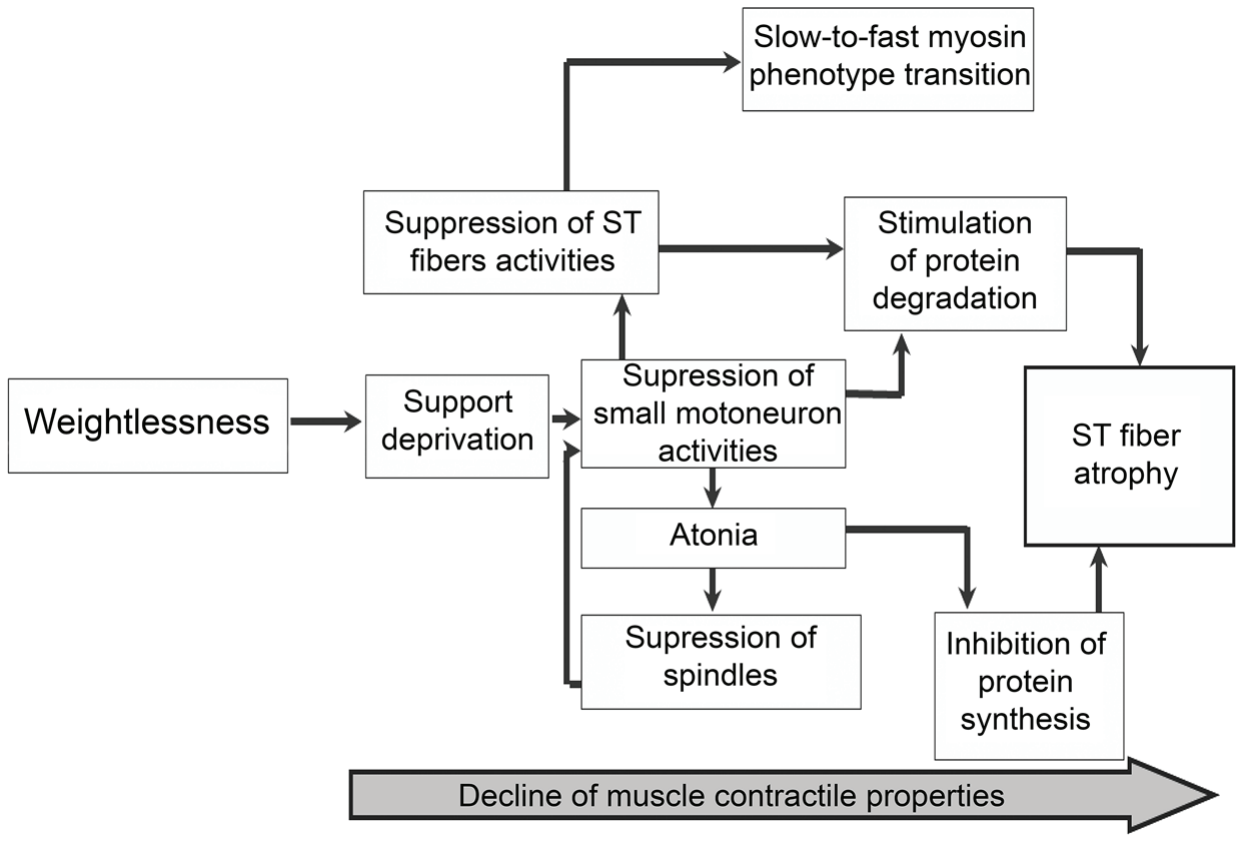
The hypothetical scheme of support withdrawal consequences in postural muscle. The scheme supposes that the exposure to weightlessness leads to the withdrawal of support afferentation. The support withdrawal induces the decline in slow motor unit activity and consequently the decline in mechanical activities of the slow-twitch muscle fibers. This decline means the reduced mechanical reflectory and then intrinsic muscle stiffness. The disuse of slow-twitch muscle fibers leads to reduced protein synthesis and increased protein breakdown, followed by the morphological signs of muscle atrophy. Simultaneously in disused fibers the alteration of myosin heavy chain isoforms expression pattern is followed by the slow-to-fast phenotypic transition. ST—slow-twitch.

For instance, our studies showed the crucial role of support afferentation withdrawal in muscle alterations under hypogravity. These studies corroborated our hypothesis that the withdrawal of support afferentation inactivates the slow motor units pool (Kirenskaia et al., 1986) which inevitably leads to selective inactivation, and subsequent atony and atrophy, of muscle fibers expressing the slow isoform of myosin heavy chain (which constitutes the majority of soleus muscle fibers) (Figure 3; Shenkman et al., 2004). Fibers that have lost a significant part of cytoskeletal molecules (Litvinova et al., 2004; Shenkman et al., 2004) are incapable of effective actomyosin motor mobilization (Litvinova et al., 2004; Ogneva et al., 2011a,b) which leads to a lower calcium sensitivity and lower range of maximal tension in calcium-induced contraction. Support withdrawal also leads to lower efficiency of protective mechanisms (NO synthase) and decreased activity of AMP-activated protein kinase (Vilchinskaya et al., 2015).

Dependence of the main anabolic and catabolic signaling pathways on the state of support afferentation system is still needs to be assessed, and the immersion model seems best suited for this purpose. Also, these studies (particularly involving plantar mechanical stimulation) provide convincing evidence that it is possible to maintain the key parameters of the internal environment of muscle fibers by means of low-intensity contractile activity without substantial external resistance. Data on the state of cytoskeletal proteins as well as contractile and stiffness properties of myofibrils show that the intrinsic resistance of muscle fibers found under the conditions of normal gravity is capable of maintaining the intracellular homeostasis even at low levels of contractile activity.

Thus, “dry” immersion studies, albeit rare as compared with studies using other models of microgravity, have already considerably contributed to the gravitational physiology of skeletal muscle. Future research, no doubt, will have even greater potential.

## Author Contributions

Both authors listed have made a substantial, direct and intellectual contribution to the work, and approved it for publication.

### Conflict of Interest Statement

The authors declare that the research was conducted in the absence of any commercial or financial relationships that could be construed as a potential conflict of interest.
